# Music Modulates Cognitive Flexibility? An Investigation of the Benefits of Musical Training on Markers of Cognitive Flexibility

**DOI:** 10.3390/brainsci11040451

**Published:** 2021-04-02

**Authors:** Miriam Gade, Kathrin Schlemmer

**Affiliations:** 1 Department of Psychology, Catholic University of Eichstätt-Ingolstadt, 85072 Eichstätt, Germany; 2 Medical School Berlin, Faculty of Sciences, 14197 Berlin, Germany; 3 Department of Cultural Studies, Catholic University of Eichstätt-Ingolstadt, 85072 Eichstätt, Germany; kathrin.schlemmer@ku.de

**Keywords:** training, amateur musicians, piano students, attention

## Abstract

Cognitive flexibility enables the rapid change in goals humans want to attain in everyday life as well as in professional contexts, e.g., as musicians. In the laboratory, cognitive flexibility is usually assessed using the task-switching paradigm. In this paradigm participants are given at least two classification tasks and are asked to switch between them based on valid cues or memorized task sequences. The mechanisms enabling cognitive flexibility are investigated through two empirical markers, namely switch costs and n-2 repetition costs. In this study, we assessed both effects in a pre-instructed task-sequence paradigm. Our aim was to assess the transfer of musical training to non-musical stimuli and tasks. To this end, we collected the data of 49 participants that differed in musical training assessed using the Goldsmiths Musical Sophistication Index. We found switch costs that were not significantly influenced by the degree of musical training. N-2 repetition costs were small for all levels of musical training and not significant. Musical training did not influence performance to a remarkable degree and did not affect markers of mechanisms underlying cognitive flexibility, adding to the discrepancies of findings on the impact of musical training in non-music-specific tasks.

## 1. Introduction

Human behavior is remarkably flexible and can easily be adapted to changing goals. For instance, a person going home from work may remember that the milk got sour; consequently, he alters the way home to come past the nearby supermarket and get some milk. People do these quick adaptations so regularly that only when they fail (i.e., miss the turnover) or see others failing (i.e., older people forgetting to pick up the prescription before going to the pharmacy), they realize that cognitive flexibility is effortful and costly. This does not only hold true for everyday life but also for professional life, for instance as a musician. For example, the processes of music reading and playing require constant adaptations to be made following a change of key, time signature, tempo, or loudness shown by the conductor.

### 1.1. Musical Training and Cognitive Benefits

Recently, professions—and their inherent expertise-promotion owing to professionals often undergoing extended training—have attracted much interest in cognitive psychology. Given the concept of cognitive plasticity which refers to changes in the cognitive system because of prolonged engagement in a task such as learning to play an instrument, musicians were found to be a valuable group to study. The obvious complexity of music making and the high amount of training that professional musicians often complete [1,2], have inspired the idea of a possible transfer of musical training to other areas of achievement—most prominently to cognitive abilities. Despite the large body of research on potential associations between musical training and cognitive abilities (for a review, cf. [3]), the direction of its influence on executive functions has yet to be more thoroughly examined. Conceptually, executive functions refer to higher-order cognitive functions, such as working memory, attentional control, or inhibition of pre-potent, or habitual responses to achieve intended goals [4,5].

A review of the existing evidence obtained in correlational studies on the relationship between musical training and executive functions among children provides mixed results. Whereas [6] demonstrate positive evidence, [7] was not able to observe a relationship between the two constructs. Overall, some longitudinal and training studies with children have suggested that musical training might be the cause for improvements in children’s memory [8,9] and executive functions [10,11,12,13,14]. Finally, studies investigating neural correlates provide some evidence for an impact of musical training on brain activity, at least in children [15]. By looking at adults, [16] found evidence that adult musicians, when compared to musically untrained persons, perform better in a test involving inhibition of irrelevant conflicting auditory and spatial information. Furthermore, in a study that used unselected student samples and a more continuous measure of musical ability, no evidence for better inhibitory abilities was found, but there were some gains for working memory updating [17]. Another recent study provided positive correlational evidence for an association of higher general and verbal intelligence, working memory, and attention skills with the duration of musical practice [18]. Concluding, the current empirical evidence on the relationship between musical training and gains outside the musical domain tends to support the predicted transfer effects only partially. Rather, these effects seem dependent on the chosen empirical markers, for example, brain activity vs. behavioral measures or study sample, for example, children vs. adults.

### 1.2. Transfer Effects of Cognitive Training

Recently, a couple of studies on cognitive plasticity have found a lack of transfer of the skills acquired from training tasks to broader contexts, namely, theoretically and/or empirically close processes, functions, or even tasks (e.g., [19,20,21]). Thus, these studies have questioned the idea of plasticity of the cognitive system, rather than the effectivity of the respective material and training (e.g., [22], for a recent suggestion as to why transfer effects are so volatile). Yet, sometimes transfer effects arise, so searching for the conditions under which trained processes transfer to other functions remains a valuable empirical quest. In addition, the theoretical rationale that defends the existence of domain-general mechanisms that work independently of paradigm and material still receives support, so the topic is still debated (e.g., [23] for a task-specific approach; e.g., [24] for an approach assuming general abilities). Therefore, further research seems necessary to disentangle the respective contribution of domain-general abilities and task-specific skills on cognitive performance. In some domains of expertise, an important promise of education has been the possible impact of people’s ability to transfer their cognitive performance in one task to other areas of cognitive activity or even broader abilities (e.g., [25] for research on bilingualism and the discussion on preserved cognitive abilities with advancing age). Thus, understanding the impact of training on achievements outside the trained domain is of great value.

### 1.3. The Current Study: Cognitive Flexibility in Musicians

In our study, we assessed the cognitive flexibility in participants with different levels of musical training (e.g., lay persons without any training, except for having received music lessons in school, and students of piano master classes) using standard cognitive tasks to examine potential transfer effects from music-specific to less music-specific material. We chose the construct of cognitive flexibility as it enables engagement and disengagement in various tasks and goals and is therefore a relevant pre-requisite for successful everyday activities.

We reasoned that, if musical training enhances cognitive function, it is likely that cognitive flexibility will show broader transfer effects, because cognitive flexibility involves all core executive functions (i.e., inhibition of formerly relevant information, such as stimulus–response rules used just before, holding currently relevant task representations in working memory, i.e., keeping unfinished tasks available, and re-orienting the focus of attention to new material, for instance when switching a task [4,24,26]. Furthermore, if there actually is an influence of musical training on cognitive flexibility, the involvement of several processes in that construct suggests that people may experience a broader impact on their everyday behavior.

### 1.4. Indices of Cognitive Flexibility

We examined to what degree musical training enhances cognitive flexibility by assessing two indices of this construct, namely, switch costs as well as n-2 repetition costs. Both measures can be assessed using a task-switching paradigm (e.g., [27,28,29] for reviews). Switch costs are measured as the difference between trials in which the current task is the same as the preceding task (i.e., repetition trials) compared to trials in which the current task is different from the preceding task (i.e., switch trials). N-2 repetition costs, on the other hand, require participants to switch among three tasks. To calculate those costs, a n-2 switch of a task (i.e., sequences of type CBA, with A, B, and C denoting different tasks) is subtracted from sequences of type ABA, an n-2 repetition of the same task. N-2 refers to the task performed two-trials-earlier in the sequence that is supposed to be either different (n-2 switch) or the same (n-2 repetition) than the current task. Switch costs are assumed to reflect the proactive interference from previous task performance that hampers the updating of working memory to fulfill current task requirements (e.g., [27,30] for reviews). N-2 repetition costs, on the other hand, are assumed to reflect, at least partially, task inhibition of recently performed tasks [31,32,33]; this inhibition is employed to prevent accidental re-execution of the task-set just performed, thus enabling people to appropriately realign their attention to the task that needs to be performed.

### 1.5. Review of Literature on Cognitive Flexibility in Musicians

Evidence for a benefit of musical training for cognitive flexibility has been presented in a recent study [34]; in this cross-sectional study, the participants (i.e., pianists and various other instrumentalists) had to alternate between reading G and F clefs. Among pianists, an effect of musical training on switch costs was observed in their first year of conservatory, whereas other instrumentalists attained the same reduction of switch costs only in their fifth year.

Contrariwise, a recent report showed that musicians had larger switch costs in a less music-specific paradigm [35]. In this study, a number Stroop task [36,37] was assessed; in the task, participants had to switch between indicating the number of digits displayed on the screen (i.e., 1 or 3) and indicating the identity of the digits (i.e., 1), which were displayed in a predictable sequence with a valid cued task switch every third trial (i.e., sequences of type AAB, whereby A and B stand for two different tasks). The researchers assessed musical training with a questionnaire that asked about participants’ musical training duration and the number of weekly hours of practice. Notwithstanding, additionally to the differing task (i.e., music-specific vs. non-music-specific), the switching frequency between [34,35] was also different, which might add to explaining the diverging results.

More comprehensively, [38] investigated the relationship between musical training and cognitive control processes (i.e., executive functions). The authors assessed six tasks, of which we chose to focus on only two of the five switching tasks to make our point hereinafter. These tasks comprised an auditory task switching paradigm as well as a visual task switching paradigm. In the auditory task switching paradigm, the tasks were pitch-height classification as well as timbre (i.e., string or wind instrument) classification—both music-specific classification tasks. In the visual task switching paradigm, which was non-music-specific, participants had to alter between a letter and a digit classification task following a pre-cued alternating runs procedure (i.e., sequences of type AABBAABB [39]). Musical training was assessed using the Ollen Musical Sophistication Index [40]. Like [35], musical training was not significantly related to switching ability in both tasks (i.e., auditory or visual). However, musical training was significantly related to the assessed working memory task, and there was a tendency for participants with higher musical training to show a worse performance in the auditory task switching paradigm, which was not expected. Altogether, the existing studies report transfer effects only for music-specific material and they report no transfer to different switching frequencies or material. However, musical training seems to benefit working memory.

At the time of this research, the existing studies had assessed only switch costs, and these might not reflect all mechanisms involved in successful cognitive flexibility. Additionally, Ref. [17,38] suggested that the aforementioned benefits of musical training are bound to working memory involvement; still, in studies using cued task switching paradigms in which participants are informed about the upcoming task by valid cues, working memory impact is not explored because people do not need to rely on their working memory to keep track of the task sequence.

### 1.6. The Present Study: Measurements and Aims

To complete the picture of the influence of musical training on cognitive flexibility, we conducted a task-switching study that was based on a memorized task sequence to maximize working memory involvement; in addition, this paradigmatic choice allowed us to assess not only switch costs, that are costs arising when the current task is different from the task just performed one trial earlier (n-1), but also n-2 repetition costs, referring to costs observed when switching back to a task performed two trials earlier (n-2).

As switch costs have also been proposed to reflect the inertia of memory in that old information prevents the processing of new information [27,41,42,43,44] rather than executive processes, we opted for n-2 repetition costs as a further measure of underlying cognitive flexibility. So far, and to the best of our knowledge, no investigation has been conducted to examine the inhibitory processes of cognitive flexibility by measuring n-2 repetition costs in a sample differing in musical training.

Thus, the present study, in which participants had to switch among three tasks, had two aims. First, we wanted to investigate the influence of musical training on n-2 repetition costs. We speculated that one of the reasons for the appearance of larger switch costs among musicians in [35] might be a greater reliance of their participants on inhibitory processes. Namely, musicians may employ more inhibition to suppress irrelevant tasks and successfully switch to new tasks. Consequentially, this group might suffer more from residual inhibition if they are required to switch back to a recently inhibited task.

**Figure 1:**
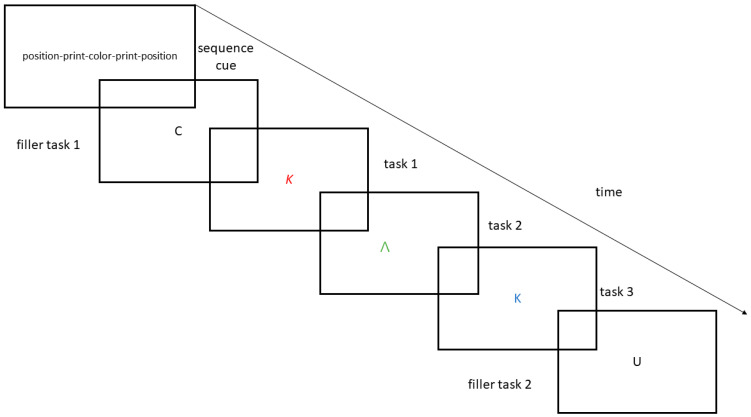
Schematic illustration of the used paradigm.

Second, we aimed to test the influence of musical training on task switching in a highly controlled paradigm with pre-instructed sequences that comprised all task transitions (i.e., conditions) of interest (i.e., sequences such as ABA, CBA, and finally, CAA; A, B, and C stand for different tasks). That is, in our paradigm, each trial comprised a mini-sequence of five tasks (see Figure 1). The first and the last task were filler tasks, tasks that were not analysed but instead used to get people started and mark the beginning and the end of a sequence. Those filler tasks had to be performed on a stimulus set different from the three tasks in between them. We included filler tasks to minimize restart costs [45]. Restart costs arise at the beginning of the engagement with a new task and have been shown to increase switch costs potentially and artificially. Furthermore, we chose to utilize a sequence of three tasks because this specific number has been shown to not exceed healthy young adults’ working memory capacity (i.e., participants would be able to remember the three tasks [46]), even if people’s working memory capacity might be susceptible to individual differences, and further increased by musical training [38].

To investigate these potential differences in working memory, we ensured that each stimulus would require a different response from participants, as these responses were dependent on the task (i.e., all stimuli were incongruent). To further illustrate this concept, we explain the stimuli hereinafter: Participants had to respond to the color of the stimulus by a 3-choice response mapping, namely, blue had to be responded to by pressing the left pointing arrow; red by pressing the down pointing arrow; and green by pressing the right pointing arrow. Likewise, the print (i.e., italics, bold, and normal) and the script (i.e., Latin, Cyrillic, or Greek) had to be responded to in a comparable, arbitrary manner. Through this procedure, stimuli were in accordance with the principle that each feature (i.e., color, print, or script) was assigned to a different response (i.e., the color of the stimulus required the left pointing arrow, the print of the stimulus required the down pointing arrow, and the script of the stimulus required the right pointing arrow). This stimulus characteristic allowed for a closer look at the type of errors participants might commit, as each keypress is indicative of the task that participants had loaded to their working memory. We predicted that higher musical training results in better sequence memory (i.e., fewer errors) and fewer accidental task repetitions, as musical training has been shown to benefit working memory performance [38]. Next, we sampled participants from a wide range of musical abilities to be able to detect differences when using a continuous measure of musical ability. Regarding the influence of musical training on our assessed indexes of cognitive flexibility, we reasoned that if musical training increases cognitive flexibility owing to better inhibition of competing tasks, we would observe larger n-2 repetition costs and, consequentially, larger switch costs. It is important to note that our task was designed to measure cognitive flexibility, not fine motor skills. Accordingly, we asked participants to use computer keyboards in our paradigm because it is a very simple motor task in which we do not assume musicians to have specific advantages. Instead, we assumed that if any differences were to appear, these would be generated by higher cognitive functions, which are the ones necessary for solving the paradigm we devised and applied.

## 2. Materials and Methods

Ethic approval: The study was approved by the Ethic Committee of the Faculty of Philosophy and Education, Catholic University of Eichstätt-Ingolstadt (2016/15).

### 2.1. Participants

Forty-nine participants took part in the experiments (31 women, mean age 21.6 years (SD = 3.4 years), with 42 right-handed). All were students at the Catholic University of Eichstaett-Ingolstadt or at the University of Music and Performing Arts Munich (HMTM). Students received either course credit or were paid 8 Euro per hour for their participation. The students of the HMTM were all attending piano master classes. The reason for choosing pianists as musically trained participants was to ensure comparability between our study and that of [34], and the fact that pianists seem to be particularly used to musical task switching; for example, they are often required to rapidly alternate between reading bass and treble clefs.

Musical training was assessed with the Goldsmiths Musical Sophistication Index (Gold-MSI, [47]) using five questions of the musical training scale and two questions concerning active engagement. The questions regarding active engagement were (1) the number of musical events visited in a year and (2) daily time spent listening to music, however those questions were not considered in the present analyses. The questions from the training scale were (1) the number of instruments played, (2) the number of hours practiced in a week during peak performance, (3) the years of instrumental training, (4) the years of music theory training, and (5) regular daily practice; each of these questions was measured by a 7-point Likert-type scale. Overall, seven items were taken out of the rather broad questionnaire [47].

We calculated participants’ overall musical training scores by summing up the scores from each of the five items to assess musical training. In our sample, we obtained a mean of 19.2, SD 9.3, range 5 to 34, suggesting a moderate level of musical training.

### 2.2. Stimuli and Task

Participants performed simple classification tasks on perceptual stimuli (see Figure 1). Those were a color classification (green vs. red vs. blue), a print classification (bold vs. normal vs. italic) and a script classification (Latin vs. Greek vs. Cyrillic). These were presented in random order in short sequences of five tasks each (see Figure 1). The first and the last task required participants to judge a letter regarding its position in the German alphabet (beginning (C, D, E), middle (N, O, P) or end (U, V, W)). Participants responded to all classifications by using the left-pointing, down-pointing, and right-pointing arrow keys on a standard German QWERTZ keyboard. Stimulus response mapping was fixed across the experiment and participants. The mapping was as follows: the left arrow key was associated with red, italics, and Cyrillic; the down arrow key was associated with green, normal, and Greek; and the right arrow key was associated with blue, bold, and Latin.

Stimuli comprised the letters K, Λ, and Φ (for Latin, Greek, and Cyrillic), which could be printed in normal, bold, or italic, and colored red, green, or blue; together, these amounted to a stimulus set of six different stimuli, which were created owing to the constraint that all stimuli had to be incongruent (i.e., requiring a different response dependent on task context, that is for example left for color, right for print, and down for script). Stimuli were written in Calibri 80 points and saved as jpegs with 96 DPI and a size of 1280 × 720 pixels.

### 2.3. Procedure

Participants were tested in a single session and used group-lab desktop PCs. The PCs were running Windows 7 and were connected to 19” flat screen monitors (with a 1280 × 1024 pixel resolution) and standard German QWERTZ keyboards via a USB connection. The rooms in which the sessions took place were located either at the Ludwig-Maximilians University of Munich or at the Catholic University of Eichstätt-Ingolstadt. The viewing distance was about 60 cm. Experiments were programmed in Eprime 2.0 Professional [48].

After signing informed consent, participants entered their demographic data into the software and started the experiment. The instructions were originally displayed on the monitor, but they could also be explained orally upon request. The stimulus–response mapping sheet was placed next to the participants. Each trial contained one sequence of five tasks—the first and the last task always required a letter-position judgement, whereas the other three tasks comprised the print, color, and script judgement tasks, which appeared in pseudo-random order. Participants could take as much time as they needed to memorize the sequence; once they hit the space bar of the keyboard, the trial began.

Then, the target stimuli appeared successively, and each stimulus remained on the screen until a response was given or 5 s had elapsed. In case of response omission, the trial was coded as an error. The first twenty trials were considered as practice, and feedback was given only at this stage. Immediately after responding, the next stimulus appeared (i.e., response stimulus interval of 0 ms). At the end of the five-task sequence, a new sequence was initialized. The three-task sequences (i.e., not the first nor the last task) were constrained to belong to one of the following types: either an n-2 repetition (i.e., color–print–color), an n-1 repetition (i.e., print–script–script), or an n-2 shift (i.e., color–print–script). Thus, the fourth task in each sequence determined to which condition (n-2 repetition, n-2 switch, or n-1 repetition) the actual sequence belonged and was therefore the only task analysed.

All sequences (for each sequence, 78 occurrences were included) and single tasks occurred equally often throughout 12 blocks of 20 trials. After each block, participants were invited to take a short break; they started the next block by pressing the space bar. Stimuli were selected in a way to ensure that only one dimension was assigned to each response key (i.e., incongruent stimuli), and stimulus repetition was excluded.

During the experimental blocks, we asked participants to rate their own performance on a 7-point Likert-type scale. They had to judge how well they performed in the last sequence; how well they prepared for the last performed sequence; and how difficult it was. The time points in which these questions occurred were randomly selected, and questions were never repeated on two consecutive trials. Within each block of 20 trials, participants were asked to evaluate their performance three times. However, since this paper focuses on the differential influences of musical training on cognitive flexibility, these data were not included in our statistical analyses and are reported in Appendix A. At the end of the experiment, all participants were asked to provide information about their musical training by seven items taken from the Gold MSI. Overall, the experiment took about 75 to 90 min.

### 2.4. Design

These experiments were designed to investigate the impact of musical training on two differential measures of cognitive flexibility, namely, switch costs and n-2 repetition costs. Thus, participants’ performance in the fourth task of a mini sequence was either classified as n-2 switch, n-2 repetition, or n-1 task repetition (i.e., condition). The dependent variables were reaction times (RTs) and error rates (ER). Furthermore, we split up errors by error types and analysed % occurrence of erroneous task repetitions.

### 2.5. Data Trimming

We only analysed participants’ performance on every fourth task of the mini sequence, and analyses were conducted by condition (i.e., n-2 switch, n-2 repetition, or n-1 repetition). To this end, for RT analysis, we considered only the trials in which the second, third, and fourth tasks were performed correctly (i.e., 87.4% of the collected data). Further, data trimming removed all RTs that were either faster or slower than the mean RT by 3 standard deviations (i.e., either 3 plus or 3 minus) in each participant per condition (i.e., n-2 switch, n-2 repetition, and n-1 repetition; 2% of RTs were removed in this data trimming procedure). Furthermore, the RTs were transformed by log10 to reduce the influence of overall slower participants. On average, the number of kept task sequences (with the maximum possible being 78 for all task sequences) were 67 for n-2 repetitions (range 40 to 75), 69 for n-2 switches (range 38 to 76), and 68 for n-1 repetition (range 52 to 76). Switch cost analysis included conditions of type CBA and CAA only, whereas n-2 repetition costs analysis comprised only conditions of type CBA and ABA.

Concerning error analysis, we ensured that the errors on the fourth task were not influenced by former errors by retaining only trials in which the second and third tasks were performed correctly (i.e., 8.5% of the collected data). Error rate was analysed with the same mixed effects model as RT data, using an underlying binomial distribution. Regarding the error type analysis, we analysed the erroneous responses in the fourth task of the sequence by its actual stimulus and the given response dependent on condition. In particular, we were interested in the proportion of accidental task repetitions and how these were modulated by musical training.

Data were analysed in linear mixed effects models in order to preserve a maximal amount of information. We predicted the RT using condition (for switch costs: n-1 repetition vs. n-2 switch, and for n-2 repetition costs, n-2 repetition vs. n-2 switch) and participants’ musical training as fixed factors, and the participant variable was entered as a random intercept. Condition was additionally modeled as random slope. Data were analysed using R 4.03 [49], the “lme4” package [50]. Data and analysis code can be found at https://osf.io/5chwa/.

## 3. Results

### 3.1. Switch Costs

In our first analysis, we investigated the influence of musical training on the size of switch costs. The obtained t-values, coefficients, and standard errors for fixed effects can be found in Table 1 for RT data and in Table 2 for error data. Marginal R^2^ which denotes variance explained by fixed effects (switch costs, musical training) was only 0.147, conditional R^2^ that also includes random effects (switch costs, participant) variance was 0.279. Random effect variance was 0.003 for the intercept (participant) and 0.002 for the slope (condition). Please note that data were log-transformed.

Overall, differences between CAA and CBA trials were present, at an average of 304 ms (SD 177 ms). Musical training had no significant influence on mean RT at all (see Table 1) and did not affect switch costs (see Table 1).

**Figure 2:**
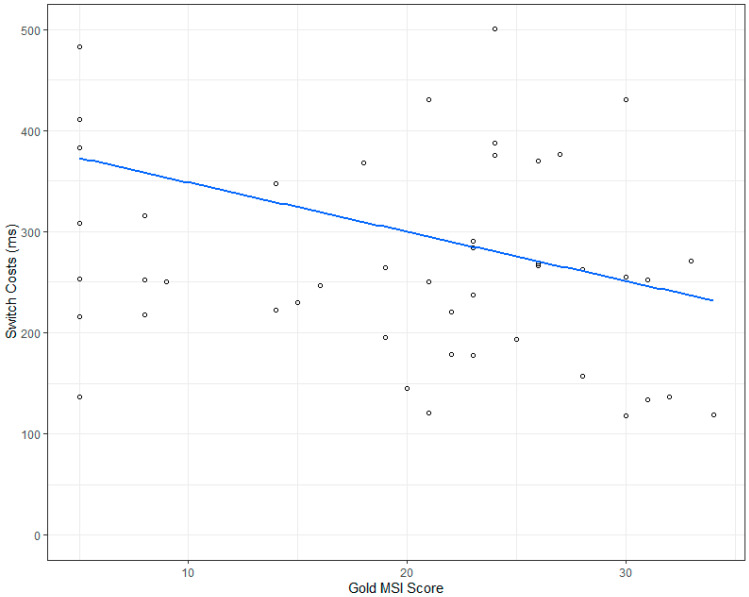
Switch costs (ms) by Gold MSI score obtained. The blue line is the fitted regression line.

Analysing the error data, we did not observe any switch costs (see Table 2), any influence of musical training, nor any modulation of switch costs by musical training. Table 3 shows descriptive RT data and error rates by condition, and Figure 2 shows a graphical depiction of the RT data for switch costs.

### 3.2. N-2 Repetition Costs

Our second analysis focused on the influence of musical training on the size of n-2 repetition costs; overall, we did observe n-2 repetition costs, but they amounted to only 8 ms (SD 17, see Table 3 and Table 4). Marginal R^2^, which denotes variance explained by fixed effects (n-2 repetition costs, musical training) was only 0.015, conditional R^2^ that also includes random effects (participants) variance was 0.161. Random effect variance was 0.003 for the intercept (participant) and <0.001 for the slope (condition). Please note that data were log-transformed.

However, we did observe an influence of musical training on mean RT (see Table 4) but no interaction between n-2 repetition costs and musical training.

**Figure 3:**
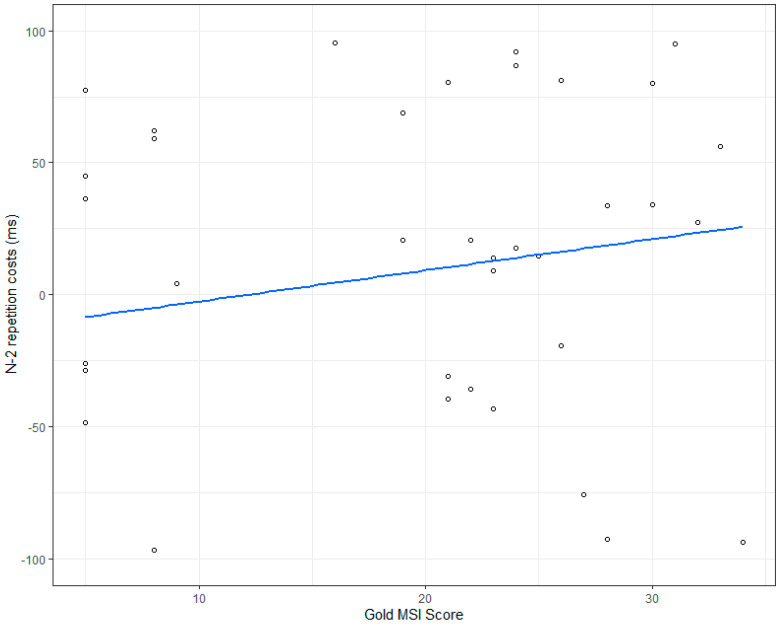
N-2 repetition costs (ms) by Gold MSI score obtained. The blue line is the fitted regression line.

For the error data (see Table 5), we did not observe any influence of n-2 repetition costs, any influence of musical training, nor any modulation of n-2 repetition costs by musical training. Table 3 shows descriptive RT data and error rates by condition and Figure 3 shows a graphical depiction of these data for n-2 repetition costs.

### 3.3. Proportion of Erroneous Task Repetitions by Musical Training

Among all errors committed in the fourth task of the sequence, the overall occurrence of erroneous task repetitions was 45.4%. Once again, we did not observe any influences of the n-2 switches or n-2 repetition sequences on the occurrence of erroneous task repetitions (*t*(41) = 0.484, *p* = 0.629)); musical training did not influence the occurrence of erroneous task repetitions (*t*(41) = 0.599, *p* = 0.551)); and conditions were not influenced by musical training (*t*(41) = 0.036, *p* = 0.973)). Please note that not all participants committed errors in the fourth task and therefore degrees of freedom were altered.

## 4. Discussion

In our study we assessed the influence of musical training on two markers of the processes underlying cognitive flexibility. We provided participants with an experimental setting in which they were required to perform small sequences of five tasks. Although the first and the last tasks were filler tasks (i.e., performed on a different stimulus set that was not analysed), participants’ accuracy in these filler tasks reached ratios of 97% for both positions. In this study, we used pre-instructed task sequences because they were presumed to maximize the positive influence of musical training on working memory [38] in addition to assessing a sample with more variance in musical ability (i.e., novices and experts). Additionally, we used non-music-specific tasks to investigate the transfer effects of musical training into broader domains. We assessed musical training by five items from the Gold MSI [47] training scale; our study participants had a score range of 5 to 34 (i.e., maximum sum of 35 points given the selected items).

Our results showed that we observed switch costs but no significant n-2 repetition costs. Moreover, we did not observe any influence of musical training on switch costs, see Figure 2 or on n-2 repetition costs (see Figure 3).

We hypothesized that the small size of n-2 repetition costs was due to two different causes. First, recent studies have questioned the reliability of this measure, suggesting that the effect occurs by chance [36,51]; thus, these studies highlight that it should, at least, not be used when assessing individual differences. Second, numerous experimental features have been shown to influence the size of n-2 repetition costs, among which chunking and explicit sequence knowledge have been observed [52]. Koch and colleagues [52] let participants work on small sequences of six tasks, and they analysed data from 16 participants who were able to report four items (out of the six-task sequence); their findings showed that n-2 repetition costs in a condition that arranges tasks sequentially were significantly reduced compared to a condition in which no sequence underlies task performance. Still, although their participants showed reduced n-2 repetition costs (i.e., from 147 to 32 ms), they were not completely absent.

In the explicitly instructed task sequences used in the present study, n-2 repetition costs were even further reduced, supporting the notion that n-2 repetition costs are influenced by explicit sequence knowledge. Although Koch and colleagues [52] advocate that assembling task representation into chunks of three items (i.e., mainly because they have a six-task sequence) may have an impact on study results, this strategy was not applicable to the present study given the used sequences only comprised three tasks. Therefore, it seems that explicit knowledge about an n-2 repetition might be sufficient to alter the balance between task activation and inhibition, namely, tilting the balance toward task activation and reducing the need for task inhibition even more [33,53].

Interestingly, this change in task activation was not affected by musical training. Although we initially speculated that using a to-be-memorized task sequence could boost the performance differences between participants with varying levels of musical training, we observed no such differential influence throughout the analyses. That is, neither the size of switch costs nor the size of n-2 repetition costs was influenced by musical training in both reaction times and error rates, only mean reaction times was. We hypothesize that these results are due to our methodological approach of employing repeated tasks that are known to reduce n-2 repetition costs [31]; maybe the experimental design was not sensitive enough, leading to an inability to reveal the benefits of musical training for the measure of inhibition employed in this study, namely the n-2 repetition costs. Likewise, our sample was of rather small size compared to other individual differences studies, although we included a highly proficient group, but effects were not detectable [38].

Does this imply that musical training does not influence cognitive flexibility as measured by n-2 repetition costs or by switch costs? We argue that, in our sample, musical training was quite skewed—the median was 22 (i.e., quite high, given the scale ranges from 5 to 35) and the lowest score was five. This means that all participants had at least some musical training. On this topic, a previous study showed that cognitive flexibility differences level off quickly once a certain training level is reached [34], and this assumption may explain our results. Furthermore, although cognitive flexibility is often subsumed under the term “executive functions” (i.e., the functions necessary for goal-directed behavior), there are paradigms other than task switching that might be more sensitive to differences in samples that have been subject to prolonged training, such as dual tasking, e.g., the performance of two tasks in close temporal sequence that require planning and hierarchical structuring of task performance [54].

Looking at existing studies using task switching training, it has been found that switch costs were hardly reduced even after prolonged training sessions [55], and that the cognitive functions that benefit most from training are task-set retrieval and scheduling. Those functions refer to configurations of the cognitive system more generally and do not directly target the origin of switch costs. Instead, they can be regarded as adjustments to the experimental situation of rapidly switching between different tasks by increasing the attentional focus and episodic memory formation [56]. Currently, the theories addressing the origin of switch costs highlight memory processes (e.g., formation of episodic memory traces of successful task completion and henceforth the overcoming of proactive interference from those competing memory traces to complete current task demands). This overcoming of interference might include higher-order task inhibition. This reasoning presupposes episodic retrieval and proactive interference causes for the observed costs, rather than processes of rapid task-set reconfiguration in case of a task switch. Nevertheless, a theoretical integration of such memory processes into the construct of executive functions has yet to be conducted (see [23,30] for first attempts).

This study had some limitations. First, we did not assess other well-known influential factors of training gains, such as socio-economic status [17,57] or working memory capacity [58,59]. However, our sample exclusively comprised university students who came from the same region, suggesting that it was somewhat homogenous regarding intelligence, working memory capacity, and socio-economic factors. Second, although recent studies comparing children receiving musical training to those not receiving such training [15] suggest that transfer effects are present in neural correlates (i.e., neuronal efficiency), we only utilized behavioral measures in our study. Thus, our study methodology was probably less sensitive to subtle changes in the underlying brain structures. Third, we used students who were at the peak of their cognitive performance, and based on a previous study [60], we acknowledge that the inherent characteristics of our sample might have further leveled off these aforementioned subtle differences. Finally, since research suggests that transfer might be limited mainly to music-related material, a promising endeavor for further research might be to include music-related as well as music-unrelated transfer tasks to examine if and how transfer decreases with increasing distance to musical relatedness.

## 5. Conclusions

In conclusion, our study found no evidence of a connection between students’ cognitive flexibility and musical training when switching between tasks based on a memorized sequence. Our results suggest that the benefits of musical training are rather volatile and not so easily transferred to non-music-specific tasks if those do not tackle other components of executive functions such as working memory [17,38]. In light of the literature, we emphasize that, although musical training might be beneficial for music-specific tasks, such training may not be easily generalizable to non-music-specific stimulus material.

## Figures and Tables

**Table 1:** Estimates for the mixed model on switch costs (dependent variable: reaction times).

Factor	Estimate	Standard Error	DF	*t*	*p*
Intercept	2.99	0.02	47.2	164.61	<0.001
Musical training	<0.001	0.00	47.08	−0.89	0.38
Condition (n-2 switch vs. n-1 repetition)	0.14	0.02	47.13	7.87	<0.001
Musical training × condition	<0.001	0.00	46.89	−1.55	0.13

Note. DF denotes degrees of freedom.

**Table 2:** Estimates for mixed model on switch costs (dependent variable: error data).

Factor	Estimate	Standard Error	*z*	*p*
Intercept	3.31	0.33	9.9	<0.001
Musical training	0.02	0.02	1.18	0.24
Condition	−0.07	0.34	−0.2	0.84
Musical training × Condition	−0.01	0.02	−0.64	0.52

**Table 3:** Descriptive Values for mean reaction times (RT) in ms (SD) and error rates (ER) in percent (SD) for three conditions.

Condition	n-1 Repetition	n-2 Switch	n-2 Repetition
RT (SD)	1002 (133.5)	1306 (210.6)	1314 (186.7)
ER (SD)	3.2 (2.0)	3.9 (3.6)	5.0 (5.3)
Switch Costs (RT)	304 (175.3)	n-2 Repetition Costs (RT)	8 (115.8)
Switch Costs (ER)	0.7 (3.9)	n-2 Repetition Costs (ER)	1.1 (3.8)

**Table 4:** Estimates for mixed model on n-2 repetition costs (dependent variable: reaction times).

Factor	Estimate	Standard Error	DF	*t*	*p*
Intercept	3.13	0.02	46.92	166	<0.001
Musical training	<0.001	0.01	46.86	−2.03	0.05
Condition (n-2 repetition vs. n-2 switch)	<0.001	0.00	46.17	0.05	0.72
Musical training × condition	<0.001	0.00	45.77	−0.51	0.46

Note. DF denotes degrees of freedom.

**Table 5:** Estimates for mixed model on n-2 repetition costs (dependent variable: error data).

Factor	Estimate	Standard Error	*z*	*p*
Intercept	3.02	0.34	8.98	<0.001
Musical training	0.01	0.02	0.76	0.45
Condition	0.21	0.29	0.74	0.46
Musical training × condition	<0.001	0.01	−0.29	0.77

## Data Availability

Data and analysis code can be found at https://osf.io/5chwa/ (accessed on 30 March 2021).

## Appendix A

Analyses of self-rated performance

Performance could be rated from very good (1) to very bad (7); preparation to perform the instructed sequence was rated from very good (1) to non-existent (7); and sequence difficulty from very easy (1) to very difficult (7). Mean ratings were 2.84 (SD = 0.99) for the first question, 2.54 (SD = 0.83) for the second question, and 2.85 (SD = 0.87) for the third question, suggesting that participants perceived their performance as good, that they sensed they were prepared, and that they did not find the sequences very difficult.

Ratings in the first and third questions were affected by condition (*t*(48) = 2.526, *p* = 0.026, *t*(48) = 2.534, *p* = 0.012)), but musical training had no influence on mean rating and did not modulate the rating dependent on condition for any question (all *t*s < 1.5, all ps > 0.14).

## References

[1] Ericsson K.A., Krampe R.T., Tesch-Römer C. (1993). The role of deliberate practice in the acquisition of expert performance. Psychol. Rev..

[2] Platz F., Kopiez R., Lehmann A.C., Wolf A. (2014). The influence of deliberate practice on musical achievement: A meta-analysis. Front. Psychol..

[3] Schellenberg E.G., Hallam S., Cross I., Thaurt M. (2016). Music training and nonmusical abilities. The Oxford Handbook of Music Psychology.

[4] Miyake A., Friedman N.P., Emerson M.J., Witzki A.H., Howerter A., Wager T.D. (2000). The Unity and Diversity of Executive Functions and Their Contributions to Complex “Frontal Lobe” Tasks: A Latent Variable Analysis. Cogn. Psychol..

[5] Smith E.E. (1999). Storage and Executive Processes in the Frontal Lobes. Science.

[6] Degé F., Kubicek C., Schwarzer G. (2011). Music Lessons and Intelligence: A Relation Mediated by Executive Functions. Music. Percept. Interdiscip. J..

[7] Schellenberg E.G. (2011). Examining the association between music lessons and intelligence. Br. J. Psychol..

[8] Degé F., Wehrum S., Stark R., Schwarzer G. (2011). The influence of two years of school music training in secondary school on visual and auditory memory. Eur. J. Dev. Psychol..

[9] Roden I., Grube D., Bongard S., Kreutz G. (2013). Does music training enhance working memory performance? Findings from a quasi-experimental longitudinal study. Psychol. Music.

[10] Bugos J.A., Demarie D. (2017). The effects of a short-term music program on preschool children’s executive functions. Psychol. Music.

[11] Frischen U., Schwarzer G., Degé F. Der Einfluss von Musiktraining auf Exekutive Funktionen im Vorschulalter—Rhythm is it? In Proceedings of the Jahrestagung Deutsche Gesellschaft für Musikpsychologie, Eichstaett, Germany, 7–8 September 2019.

[12] Jaschke A.C., Honing H., Scherder E.J.A. (2018). Longitudinal Analysis of Music Education on Executive Functions in Primary School Children. Front. Neurosci..

[13] Moreno S., Bialystok E., Barac R., Schellenberg E.G., Cepeda N.J., Chau T. (2011). Short-Term Music Training Enhances Verbal Intelligence and Executive Function. Psychol. Sci..

[14] Bolduc J., Gosselin N., Chevrette T., Peretz I. (2020). The impact of music training on inhibition control, phonological processing, and motor skills in kindergarteners: A randomized control trial. Early Child Dev. Care.

[15] Zuk J., Benjamin C., Kenyon A., Gaab N. (2014). Behavioral and Neural Correlates of Executive Functioning in Musicians and Non-Musicians. PLoS ONE.

[16] Bialystok E., DePape A.-M. (2009). Musical expertise, bilingualism, and executive functioning. J. Exp. Psychol. Hum. Percept. Perform..

[17] Okada B.M., Slevc L.R. (2018). Individual differences in musical training and executive functions: A latent variable approach. Mem. Cogn..

[18] Criscuolo A., Bonetti L., Särkämö T., Kliuchko M., Brattico E. (2019). On the Association Between Musical Training, Intelligence and Executive Functions in Adulthood. Front. Psychol..

[19] Pfister R., Schwarz K.A., Wirth R., Lindner I., Gade M., Zoelch C., Seitz-Stein K. (2017). My Command, My Act: Observation Inflation in Face-To-Face Interactions. Adv. Cogn. Psychol..

[20] Melby-Lervåg M., Hulme C. (2013). Is working memory training effective? A meta-analytic review. Dev. Psychol..

[21] Rode C., Robson R., Purviance A., Geary D.C., Mayr U. (2014). Is Working Memory Training Effective? A Study in a School Setting. PLoS ONE.

[22] Meiran N., Dreisbach G., Von Bastian C.C. (2019). Mechanisms of working memory training: Insights from individual differences. Intelligence.

[23] Hommel B., Wiers R.W. (2017). Towards a Unitary Approach to Human Action Control. Trends Cogn. Sci..

[24] Friedman N.P., Miyake A. (2017). Unity and diversity of executive functions: Individual differences as a window on cognitive structure. Cortex.

[25] Bak T.H., Nissan J.J., Allerhand M.M., Deary I.J. (2014). Does bilingualism influence cognitive aging?. Ann. Neurol..

[26] Miyake A., Friedman N.P. (2012). The Nature and Organization of Individual Differences in Executive Functions. Curr. Dir. Psychol. Sci..

[27] Kiesel A., Steinhauser M., Wendt M., Falkenstein M., Jost K., Philipp A.M., Koch I. (2010). Control and interference in task switching—A review. Psychol. Bull..

[28] Vandierendonck A., Liefooghe B., Verbruggen F. (2010). Task switching: Interplay of reconfiguration and interference control. Psychol. Bull..

[29] Meiran N. (2014). The task-cuing paradigm: A user’s guide. Task Switching and Cognitive Control.

[30] Koch I., Poljac E., Müller H., Kiesel A. (2018). Cognitive structure, flexibility, and plasticity in human multitasking—An integrative review of dual-task and task-switching research. Psychol. Bull..

[31] Gade M., Schuch S., Druey M.D., Koch I., Grange J.A., Houghton G. (2014). Inhibitory control in task switching. Executive Control and Task Switching.

[32] Grange J.A., Kowalczyk A.W., O’Loughlin R. (2017). The effect of episodic retrieval on inhibition in task switching. J. Exp. Psychol. Hum. Percept. Perform..

[33] Koch I., Gade M., Schuch S., Philipp A.M. (2010). The role of inhibition in task switching: A review. Psychon. Bull. Rev..

[34] Slama H., Rebillon E., Kolinsky R. (2017). Expertise and cognitive flexibility: A Musician’s Tale. J. Cult. Cogn. Sci..

[35] Moradzadeh L., Blumenthal G., Wiseheart M. (2015). Musical Training, Bilingualism, and Executive Function: A Closer Look at Task Switching and Dual-Task Performance. Cogn. Sci..

[36] Rey-Mermet A., Gade M., Oberauer K. (2018). Should we stop thinking about inhibition? Searching for individual and age differences in inhibition ability. J. Exp. Psychol. Learn. Mem. Cogn..

[37] Salthouse T.A., Meinz E.J. (1995). Aging, Inhibition, Working Memory, and Speed. J. Gerontol. Ser. B Psychol. Sci. Soc. Sci..

[38] Slevc L.R., Davey N.S., Buschkuehl M., Jaeggi S.M. (2016). Tuning the mind: Exploring the connections between musical ability and executive functions. Cognition.

[39] Rogers R.D., Monsell S. (1995). Costs of a predictible switch between simple cognitive tasks. J. Exp. Psychol. Gen..

[40] Ollen J.E. (2006). A Criterion-Related Validity Test of Selected Indicators of Musical Sophistication Using Expert Ratings. Ph.D. Thesis.

[41] Allport A., Styles E.A., Hsieh S., Umilt C., Moscovitch M. (1994). Shifting intentional set: Exploring the dynamic control of tasks. Conscious and Nonconscious Information Processing.

[42] Koch I. (2001). Automatic and intentional activation of task sets. J. Exp. Psychol. Learn. Mem. Cogn..

[43] Koch I. (2003). The role of external cues for endogenous advance reconfiguration in task switching. Psychon. Bull. Rev..

[44] Koch I., Allport A. (2006). Cue-based preparation and stimulus-based priming of tasks in task switching. Mem. Cogn..

[45] Poljac E., Koch I., Bekkering H. (2009). Dissociating restart cost and mixing cost in task switching. Psychol. Res..

[46] Cowan N. (2001). The magical number 4 in short-term memory: A reconsideration of mental storage capacity. Behav. Brain Sci..

[47] Müllensiefen D., Gingras B., Musil J., Stewart L. (2014). The Musicality of Non-Musicians: An Index for Assessing Musical Sophistication in the General Population. PLoS ONE.

[48] Psychology Software Tools (2015). Eprime 2.0 Professional.

[49] R Core Team (2013). R: A Language and Environment for Statistical Computing.

[50] Bates D., Mächler M., Bolker B., Walker S. (2014). Fitting linear mixed-effects models using lme4. arXiv.

[51] Kowalczyk A.W., Grange J.A. (2017). Inhibition in Task Switching: The Reliability of the n − 2 Repetition Cost. Q. J. Exp. Psychol..

[52] Koch I., Philipp A.M., Gade M. (2006). Chunking in Task Sequences Modulates Task Inhibition. Psychol. Sci..

[53] Philipp A.M., Koch I. (2006). Task inhibition and task repetition in task switching. Eur. J. Cogn. Psychol..

[54] Strobach T., Salminen T., Karbach J., Schubert T. (2014). Practice-related optimization and transfer of executive functions: A general review and a specific realization of their mechanisms in dual tasks. Psychol. Res..

[55] Strobach T., Liepelt R., Schubert T., Kiesel A. (2012). Task switching: Effects of practice on switch and mixing costs. Psychol. Res..

[56] Strobach T., Frensch P.A., Soutschek A., Schubert T. (2012). Investigation on the improvement and transfer of dual-task coordination skills. Psychol. Res..

[57] Morton J.B., Harper S.N. (2007). What did Simon say? Revisiting the bilingual advantage. Dev. Sci..

[58] Gade M., Paelecke M. (2019). Talking matters—Evaluative and motivational inner speech use predicts performance in conflict tasks. Sci. Rep..

[59] Shipstead Z., Redick T.S., Engle R.W. (2012). Is working memory training effective?. Psychol. Bull..

[60] Bialystok E. (2009). Bilingualism: The good, the bad, and the indifferent. Biling. Lang. Cogn..

